# Helios.TALK: A decentralised messaging framework that preserves the privacy of users

**DOI:** 10.12688/openreseurope.14421.1

**Published:** 2022-03-04

**Authors:** Ioannis Sarridis, Vasiliki Gkatziaki, Emmanouil Krasanakis, Nikos Giatsoglou, Nikos Sarris, Symeon Papadopoulos, Ioannis Kompatsiaris

**Affiliations:** 1 Information Technologies Institute, Centre for Research and Technology Hellas (CERTH), Thessaloniki, 57001, Greece

**Keywords:** communication applications, online social networks, peer-to-peer networks, instant messaging

## Abstract

Communication via digital means, such as mobile messaging applications (apps), plays an increasingly important role in everyday life. However, most messaging apps employ centralized computing principles that relinquish control of their users’ personal data to social network platform providers. Decentralization has been proposed as an alternative that provides trustworthiness and data confidentiality, but this comes at expense of fewer provided features and non-intuitive user experience. To address this issue, we hereby present two interconnected decentralized messaging tools, developed in the scope of the HELIOS platform, which can support new decentralized social networks. The first tool is a framework that supports the development of context-aware decentralized messaging apps in mobile Android devices by organically tying together many of the platform’s standalone decentralized operations. The second is a decentralized messaging app, called helios.TALK, that builds on the framework but accommodates additional design considerations from the perspective of end-users.

## 1 Introduction

The widespread use of smartphones has led to the increasing use of communication applications (apps) that play an important role in everyday life by allowing their users to exchange messages with friends and family around the globe. In the last few years, modern communication apps, such as WhatsApp, Facebook Messenger, WeChat, and Viber, not only allow individuals to exchange text, audio or video messages but also offer a variety of engaging features, such as reactions to messages, location sharing, sharing of memories, games, shopping, and money transfer, making the user experience more playful and engaging. The majority of messaging apps are based on the centralized computing paradigm, where central services are responsible for the collection and processing of user data in order to deliver the end user functionalities and services, for example via online endpoints. As a result, users transfer control of their personal data to some respective central authorities.

On the other hand, concerns over the confidentiality of communication app user data have been steeply rising, especially after publicised privacy breaches like the Cambridge Analytica scandal ^1^. Thus, in the last few years, a number of apps, such as Signal, Telegram, and WhatsApp have been developed to ensure secure communications by providing end-to-end encryption. Despite these efforts, there are still non-negligible risks to following centralised models, which have lead to the emergence of alternative Decentralized Online Social Networks (DOSNs). These are distributed systems for social networking that offer more secure environments by having little or very limited dependencies on central infrastructures. For example, this is often achieved by adopting peer-to-peer communication between devices. However, adoption of DOSNs is still limited, primarily because popular centralized counterparts offer huge user bases and a variety of features that makes them more appealing.

HELIOS is a modular peer-to-peer social media platform that intends to return control of personal data to users adopting security-by-design and privacy-by-design principles. In particular, it provides implementations that allow users to encrypt every piece of content, exchange messages (via one-on-one communication, group conversations or forums) and control who can access their data and information. The platform follows a modular architecture that lets developers build their own secure social media applications. Thus, HELIOS Apps (HApps) are applications developed by utilising the platform’s enablers, i.e. its building blocks, which are organized into core and extension modules, bearing important and auxiliary functionality respectively.

In this paper, we present the HELIOS Group Communications Service (GCS – Section 2) module built within the HELIOS platform and brings together many of its functionalities to provide a decentralized group management framework supporting different types of group conversations. Moreover, we present a fully decentralized mobile application, called helios.TALK ( Section 3), which makes use of the GCS and other HELIOS modules and demonstrates design sensibilities applicable to similar systems. The app is publicly available on the Google Play Store (see here).

## 2 Decentralized group communications service

The HELIOS GCS module (see *Software avaialbility* ^2^) offers a decentralized group management framework that supports the creation and maintenance of different types of user groups, such as private groups of only two persons or even forums. This is achieved by bringing together lower lever operations of other HELIOS modules to create one comprehensive solution. Supported operations include user role assignment and management: users can be members of multiple groups across different contexts and can simultaneously communicate with others in any number of these; forums are special types of groups that users are free to join or leave.

Communication services provided by the GCS module establish relational connections between nodes of the heterogeneous social graph, such as people and smart objects, within different social contexts. Best practices followed in the course of development, such as object-oriented programming abstract classes and interfaces and cohesive software components, have resulted to comprehensible and easily extensible code. Developers can build their own solutions on top of the GCS module’s implementation with few code base changes. The GCS module defines a number of manager components that either integrate other HELIOS modules or deploy new functionality and run locally, i.e., in the GCS instance unique to the HApp copy running on each user’s device.

**Figure 1.**
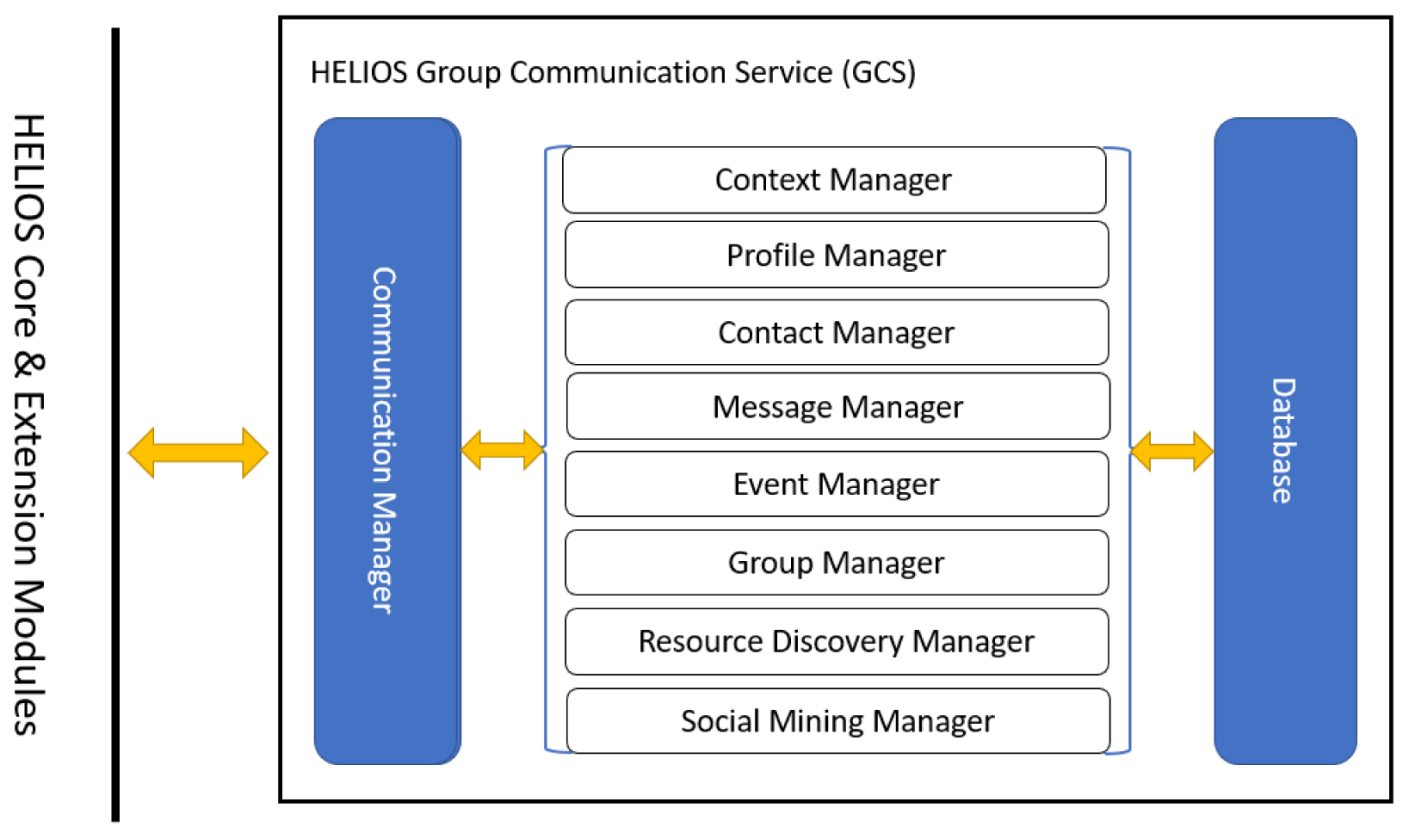
Overview of HELIOS Group Communications Service (GCS) component interactions.

Communication data are stored and retrieved from a commonly-accessed SQL (Structured Query Language) database component (maintained with the h2 library and queried through its Java DataBase Connectivity (JDBC) connector programming interface) and interactions between devices are performed only through a communication manager that interacts with all other managers. Managers work independently and interact (i.e., exchange data) with these two components, as illustrated in Figure 1. In the following subsections, we detail module component operations.

### 2.1 Communication manager

At the heart of the GCS module lies a communication manager that defines protocols for back-and-forth communication between nodes of the heterogeneous social graph based on their HELIOS identifiers, automatically assigned by the platform. Other managers rely on this interface to send and receive direct messages and manage subscriptions to HApp groups (e.g. private groups and forums) that perform multiway interactions between groups of peers. We define different message types to support these operations, which are specified in Table 1.

**Table 1.** Message types pertaining to group management.

Message Type	Description
ConnectionInfo	Connection request and respective response messages. Comprises basic user information (identifier, alias, and profile picture) and a text message.
ContextInfo	Send or respond to context invitations. Comprises user’s alias and context’s information.
MembershipInfo	Comprises information about forum members.
ForumInfo	Forum join requests or responses. Protected forums exploit this message type to notify moderators and administrators about joining requests and send back acceptance or rejection decisions.
PeerInfo	Provides information about a peer that is not included in the users’ connections but they can communicate through public/protected/secret forums.
PrivateGroupMemberListInfo	Member lists of private groups.
PrivateGroupNewMemberInfo	New private group members.
GroupInfo	Send a group invitation. Comprises user aliases and group information.
ResponseInfo	Respond to a group invitation.
Query	Send a query to search for forums, events or messages.
QueryResponse	Response to query.
Request	Request a user’s profile and response to a request.

The communication manager runs as a background service that wraps the functionality of the HELIOS core messaging module ^3^, where the latter builds upon the stack of protocols of libp2p. The supporting operations provided by the manager are critical to the development of HApps and are listed below:

**Register receivers.** Register handler functions for different types of received messages based on protocol-specific identifiers. Registering different receivers for different protocols lets developers implement different HApp operations, such as handling direct messages, friend requests, and invitations to contexts, events, groups or forums.

**Send conversation messages.** This corresponds to sending conversation messages. Messages can be sent to any HELIOS users whose identifiers are known, although HApps are expected to send messages only to alters/peers of the heterogeneous social graph. Each message comprises a header that describes their metadata and a body holding transferred data. Headers describe message types (see Subsection 2.5), unique identifiers that provide a global way of referencing messages, group identifiers that associate messages with specific conversations, groups or forums, and sent timestamps. Message bodies are serialized string representations of transferred data. For security purposes, one-on-one messages are also encrypted with the Advanced Encryption Standard (AES) protocol and the AES key is encrypted with the Rivest–Shamir–Adleman (RSA) protocol using the security & privacy module ^3^. One handler is defined that can parse all types of direct messages and needs to be registered at the receiving end with the above-described operation.

**Send invitation messages.** Invitation messages are used to let users invite others in one-on-one conversations, groups or contexts. These message headers share information describing the connection, group or context, as these are defined by the respective managers later on. One communication protocol is registered for both different invitation actions and respective responses.

**Search.** This queries searchable groups, that is, public and protected forums detailed in Table 5 of Subsection 2.7, and employs decentralized protocols to retrieve groups that match keywords or location data. Implementing this functionality requires the information exchanges described in Subsection 2.8 and two types of communication protocols (for keyword- and location- based search) are registered to support the former.

**Subscribe to groups.** GCS leverages the subscription system of the HELIOS core messaging module, in which devices listen for messages pertaining to specific groups (i.e., private groups and forums) and parse these, as long as they have access to decryption credentials that are automatically generated and shared alongside group invitations or access grants ^1^. More details about private groups can be found in Subsection 2.7. An additional listener for groups is responsible for handling events shared through the event manager of Subsection 2.6.

**Service start.** This exposes the necessary operations to start the GCS module in an application. Starting the GCS service also performs operations required to initialize other GCS managers.

**Broadcast notifications.** Notifications are broadcasted whenever an action of interest (i.e., adding and removing contacts, contexts and groups, and communication events) occurs on the GCS module’s backend, so that HApps can present appropriate information to users. This way, the GCS module facilitates user interface (UI) updates by HApps, for example to support user experience with push notifications (i.e., messages that pop up on the notification bar of mobile devices). This functionality is widely used by helios.TALK, as described in Subsection 3.1.2.

### 2.2 Context manager

HELIOS introduces the novel concept of contexts that address the heterogeneous nature of human communication dynamics. Conceptually, contexts are similar to topic-specific channels of other messaging apps, such as Slack and Discord. However, they can also be associated with locations and time frames provided by the context module. These are immutable, i.e., cannot change after context creation and are the same across context members. Regardless of these globally enforced constraints, contexts can also be activated and deactivated based on user-specific activities, as understood through specific sensor readings made available by the respective listeners of the context module ^3^. Contexts can be shared with other HELIOS users and different threads of conversations can be initiated one-on-one/group.

The GCS module makes use of the contextual ego network module’s data structure ^4^ to organize contexts and record context-specific information, such as social alters of device users and direct message events. That module assigns universally unique identifiers (UUID) to contexts upon creation. It also passes to that module additional context information that is necessary for real-world application of contexts and is responsible for keeping that information up to date. In detail, the GCS assigns public context names. These are immutable (they are assigned only once) and are visible to all members. Locally, users can also change their contexts’ viewable properties, such as names and colors; these changes are not visible to other users and can be edited at any time. Furthermore, spatial contexts can be associated with specific locations (in the form of latitude and longitude pairs and a radius) and, optionally, active timeframes detected and provided by the context module (daily, week days, weekends, weekly repetition) to define spatiotemporal contexts. These are monitored in real time using the context module . Furthermore, notifications are broadcasted when contexts become active, i.e., when both location and time constraints are met, or inactive so that frontends can listen for this type of state change.

Overall, the context manager comprises several sub-processes pertaining to disseminating contexts to other peer-to-peer network users. This is achieved through the combined effect of three sub-processes: a) a sharing context process that interacts with the communication manager to both send context invitations to alters and notify the latter of accepted and rejected invitations, b) factories that generate contexts and invitation primitives to be sent and c) an invitation receiver that listens to incoming invitation requests and facilitates efficient management of context membership. Supported context manager operations are listed in Table 2.

**Table 2.** Context manager operations.

Operation	Description
Add/remove context	Add/remove contexts to/from the database.
Add/remove context invitation	Add/remove both outgoing and incoming context invitations.
Get context(s)	Get all contexts or a specific context from the database.
Get context invitations	Get outgoing and incoming context invitations.
Handle context’s attributes	Get or set context’s public name, private name, and color.
Counters	Count the members, groups, and unread messages in a context.
Add members/conversations	Add users or groups to a context.

### 2.3 Profile manager

In alignment with common social media functionalities, users are allowed to manage their profiles, that is, how others can perceive them. Profiles are context-based so that HELIOS users can adopt different social personas in different settings and are shared with contacts. We specified the following fields that users may want to share. The alias and profile_pic fields may be sent to other users alongside in-context context invitations and are visible (i.e., are sent) to user group members adopting specific roles (e.g. forum administrators and moderators upon requesting to join and after inclusion), as explained in Subsection 2.7. The GCS module allows HApps to retrieve the profiles of connected users, for example to facilitate informative UI. The profile fields are presented in Table 3.

**Table 3.** User profile fields.

Field	Description
*contextId *	The context that profile is based on.
*alias *	The username that is visible to the user’s contacts and group member lists.
*fullname *	The user’s first name and surname.
*gender *	The user’s gender. Users can opt between male, female, and non-binary.
*country *	The user’s country.
*organization *	The user’s organization, such as university or company.
*work *	The user’s field of work.
*interests *	The user’s manually defined interests.
*quote *	The user’s quote.
*profile_pic *	The user’s profile picture.

### 2.4 Contact manager

The GCS module provides a contact manager that interacts with the database component and allows adding, removing, getting contacts or pending contacts. A pending contact factory ^2^ facilitates the generation of incoming and outgoing pending contacts, where the latter are exchanged through the communication manager. Similarly, a connection request receiver handles incoming connection requests and responses of accepting or rejecting incoming connection requests and removing outgoing connection requests.

Connection requests transfer user identifiers, aliases, profile pictures, text messages, timestamps, context identifiers, conversation identifiers, and public encryption keys. For outgoing connection requests, conversation identifiers are left empty and are filled in the request acceptance sent back to requesting peers. When establishing initial connections, context identifiers of the default context are sent. To share contexts, users send to one/more of their connections appropriate invites, using the respective tags of Table 3. If alters accept context invites, they send back connection information, including a conversation identifier corresponding to a conversation in the newly-shared context. Finally, users rejecting connection or context invitation requests respond with blank conversation identifiers.

### 2.5 Message manager

Messaging is the main functionality of the GCS module. Thus, a number of processes are developed to facilitate one-on-one and multiway communication (such as between group members) in different contexts and combine to present a message manager. Specifically, the conversation process interfaces with the database and is in control of adding incoming and sent conversation messages to a ledger of known ones, adding or removing messages in a list of favorites and raising read flags for messages shown to the user. By adding messages to favorites, one can quickly access them. Conversation messages are described by headers and bodies. Table 4 presents the supported message types. Additional processes of the message manager are responsible for sending one-on-one and group messages (through different programming endpoints), tracking the total number of incoming and unread messages, and handlers listening for incoming messages, as described in Subsection 2.1.

**Table 4.** Message types pertaining to social interactions.

Message Type	Description
TEXT	Text messages.
IMAGES	Image attachments.
FILE_ATTACHMENT	Videos or PDF files.
VIDEOCALL	Video call requests, which create video call rooms using the video call module and send participation links.
ACK	Social graph mining data exchanges (see Subsection 2.9) - these are sent alongside context information id and name.
EVENT	Share an Event.
CONTACT	Contact sharing, which sends locally stored HELIOS identifiers to recipients.
ACK_INVALID_GROUP	Remove contact requests, which are necessary for HApps to maintain integrity of local heterogeneous social networks structure when users severe heterogeneous social graph relations (e.g. “unfriend” others) in their devices.

**Table 5.** Discoverability and joining procedures of different forum types.

Forums	Discoverable	Free join	Request join	Invitation join
Public	✓	✓		✓
Protected	✓		✓	✓
Secret				✓

### 2.6 HELIOS event manager

The GCS module allows users to create and share with their connections events in different contexts. Events are described by an identifier, context identifier, title, description, location (latitude and longitude), URL, and type. The event manager interacts with the database and is responsible for creating new events, removing existing ones, and sending or responding to event invitations. Events comprise a *type* field that lets developers define their own annotations and can be shared through the messaging manager (through the *EVENT* message type), either directly with social network alters or with groups users belong to.

### 2.7 Group manager

HELIOS GCS provides two different types of groups that differ in terms of scale and control by their owner. These are: a) private groups, which can only be shared by the owner, and b) forums, which can be split into subtypes. Notably, users can be members and communicate with several groups in parallel. In this subsection, we detail all group types.

***2.7.1 Private groups*.** The main goal of private group conversations is to bring small groups of people into a single place that encourages focused communication. Although private groups bear names, they have no tags or associated spatiotemporal information and are not searchable to preserve their confidential nature. Each of them is described by a unique identifier, password, context identifier, immutable name, and owner id. All members have read and write permissions by default and only the owner can invite members to join the group. Furthermore, all group members can view identifiers, aliases, and profile pictures of other members.

The group manager’s subprocess responsible for handling private groups, interacts with the database component and provides support for creation of such groups, sending invites to other context users to join, and allowing users to leave. Finally, group members can access the group’s member list.

***2.7.2 Forums*.** Forums broadly refer to user groups that can be joined at massive scales through laxer joining procedures, such as public discoverability. To support monitoring of a large number of users, forum creators (which are by default administrators) can delegate co-administrator and moderator roles to others. Furthermore, tags and locations (these are independent from context locations) can be attached to them so that users can gain a more refined understanding of what they entail compared to reading their name only. Thus, they can request or accept invitations to join only for forums they are truly interested in. Additionally, forums are a type of social network resource and can hence be found with the resource discovery manager detailed in Subsection 2.8; this takes advantage of both names and tags to find context forums related to searched keywords. Spatiotemporal forums can also be searched based on their location given a pair of latitude and longitude coordinates.

There are three ways to join forums; a) free join, in which user requests are automatically accepted, b) request join, where administrators and moderators need to accept the users and c) invitation join, where users can be invited by any existing member and are added to forums upon accepting the invitation. Three types of forums are defined (public, protected, secret), which differ with respect to the joining procedure and discoverability, as detailed in Table 5. HELIOS Forums can be linked to specific locations within the context radius (any forum location can be selected in non-spatial contexts). In addition to their name, forums are characterized by a unique identifier, the context they belong to, a list of tags that support discovery, and a list of administrator and moderator identifiers. The GCS module provides four processes: a) *forum management* that interacts with the database component and is responsible for getting/adding/removing different types of forums, b) *forum sharing* that implements functionalities pertaining to sending and responding to forum invites, as well as requesting access to protected forums, c) a *forum factory* that is used to create new forums, and d) a forum invitation factory that is used to generate forum invites to be sent and parse incoming ones.

***2.7.3 Forum membership*.** A prevalent behaviour in online social forums, which is also expected to arise in HApps, is the emergence of unscrupulous individuals, known as trolls, who intentionally provoke other group members and disrupt conversations by diverting attention away from the original purposes of groups. Thus, the group manager needs to provide tools to HApp developers so that they let users protect themselves against this kind of behaviour. Similarly, users need to be able to address spamming and opinion spamming phenomena, which refer to the practice of -often repeatedly- delivering commercially-driven messages or organized promotion of beliefs, such as harmful misinformation. Safeguarding against potential disruptions of accepted social communication is crucial for the smooth operation of groups. Thus, the GCS module allows administrators and moderators to police group members by revoking some or all access rights from users behaving inappropriately.

To enable these measures, four member roles are defined, which differ in terms of permissions, as seen in Table 6. In an additional effort to respect privacy, not all roles have access to the member list and the latter only maintains user identifiers, aliases, and profile pictures (but not other fields maintained by profile managers). Finally, users cannot view posts of forums they have not joined, even if the latter are public; this way, moderators and administrators of even public forums can control to a certain extent which information leaks to untrustworthy individuals by revoking their read rights.

**Table 6.** Permissions of user roles. Those with access to the member list can also accept and reject user join requests.

Role	Promote/demote	Member list	Invite	Write	Read
Administrator	✓	✓	✓	✓	✓
Moderator	✓(only roles below)	✓	✓	✓	✓
Editor			✓	✓	✓
Reader			✓		✓

**Figure 2.**
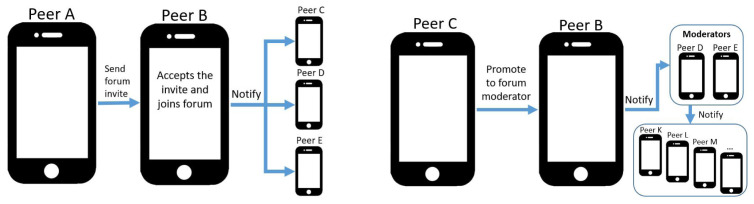
Two forum operation examples: a peer joins a forum through invitation (left), in which case they need to notify all moderators to include them in the member list, and promoting a member to moderator (right).

Users creating forums are automatically assigned administrator role and can define which role new users adopt by default when joining (with editors being the preselected value). The member list, including last known member aliases and profile pictures, is retained only by administrators and moderators, and is synchronized between them through sharing protocols. Other user roles can only obtain the list of moderator and administrator identifiers but no profile information regarding the latter. When users invite others to join forums, they send invitation messages comprising appropriate information, as depicted in Figure 2. Peers accepting invitations notify forum moderator and administrator devices to update member lists. If administrators or moderators (Peer C) decide to promote users (Peer B) from editors to moderator, they also notify the rest of forum members, as depicted in Figure 2.

### 2.8 Resource discovery manager

Resource discovery is an important function of online social networks because it allows users to search for resources through keyword queries. In decentralized networks, this function is even more challenging as the resources are not stored or known in a central location ^5^. In the GCS module, discovery is provided by the resource discovery manager, which allows searching for query-able resources, namely public and protected forums described in Subsection 2.7. 

**Figure 3.**
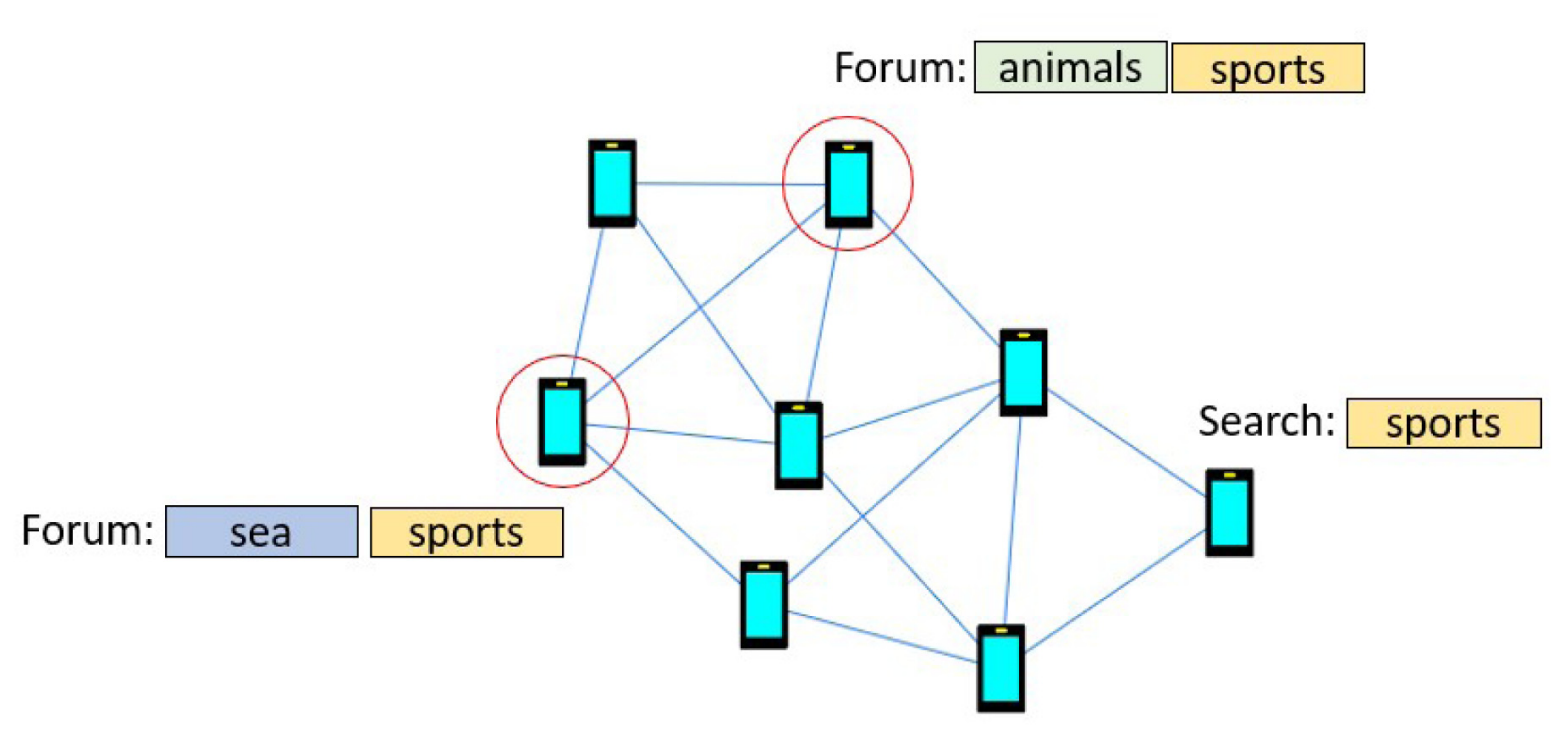
Example search in the HELIOS social graph, where a user queries the p2p network about “sports” and needs to discover related forums stored in the social graph.

Discovery does not rely on the publish-subscribe peer-to-peer substrate between users that are registered in the same forums by the group manager so that users holding resources relevant to queries are obfuscated and thus cannot be contacted directly. Instead, queries are relayed across the connected users in the heterogeneous social graph, a function that is known in the literature as unstructured peer-to-peer search ^6^. The initial design of the resource discovery manager is based on principles of privacy and bandwidth efficiency. Figure 3 depicts a search example.

The resource discovery manager supports keyword-based forum search, which is the typical search mode in search engines that users are accustomed to ^3^. To describe forum search, we distinguish between the retrieval and the communication processes of our design. With respect to retrieval, each user maintains a keyword list of their registered forums that can be queried with small computational costs. Keywords are extracted from forum titles, as well as tags provided by creators to better characterize the topic of discussion. We stress that, respecting the privacy requirements of the HELIOS platform, users do not store information about forums of other user and, thus are not aware of forum membership of their connections.

### 2.9 Mining manager

The GCS module operates as middleware between HELIOS platform enablers and HApps. Thus, in addition to core modules of the platform, it integrates extension modules that implement decentralized variations of popular social media analysis operations. Extension modules are accompanied by under-the-hood implementations of required communication and data management protocols, thus simplifying their usage. In detail, the GCS module integrates the graph mining module ^7^ and the content-aware profiling module ^8^. The first of these takes advantage of social interactions between users of the heterogeneous social graphs to induce a context-specific understanding of their latent (i.e., unobserved) preferences. Then, it provides mechanisms that can recommend recurrent interactions, such as talking with friends with whom users may not have communicated recently but may be interested in interacting next. Recommendations are performed per context. On the other hand, the content-aware profiling module analyses local image collections to obtain profiles of user interests based on correlation of these with pre-trained visual features. These could be of interest to both users and privacy-respecting personalized advertisement, such as monetized HApps that periodically exchange collections of advertisements and display only the ones that best match user interests.

**Figure 4.**
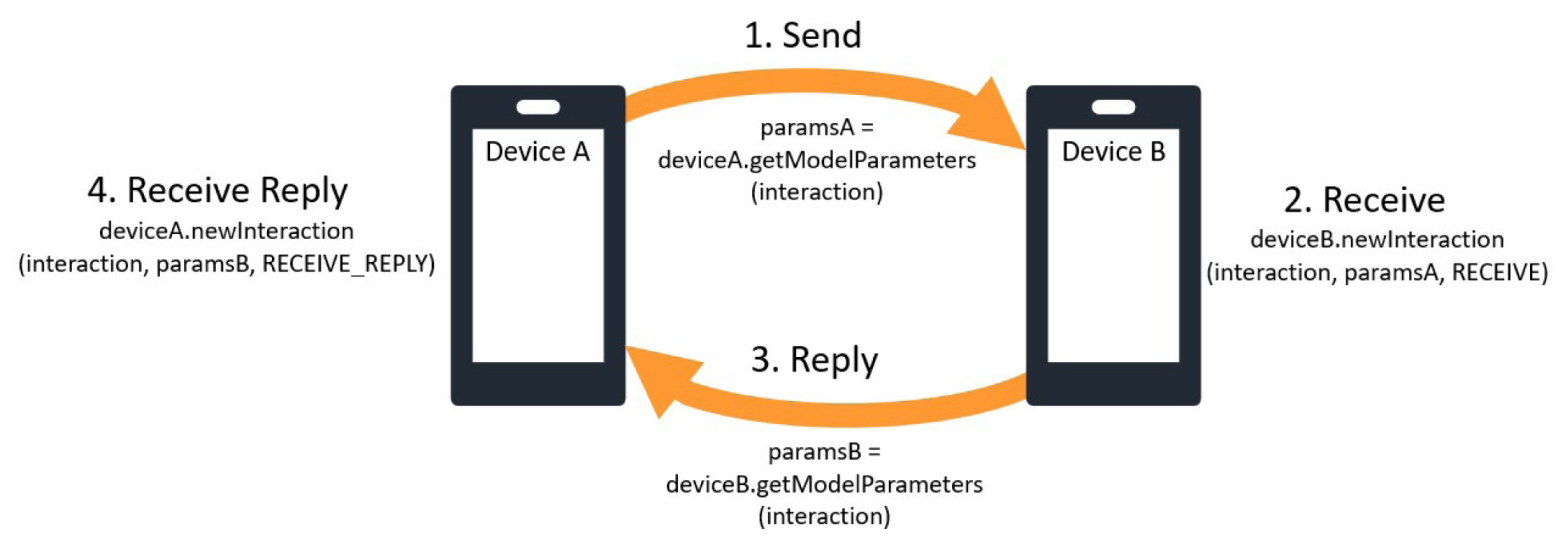
Parameter exchange scheme between graph recommendation module instances running in different HELIOS devices; device A sends mined parameters to device B and the latter replies with its own.

***2.9.1 Social graph mining integration*.** The graph mining module analyses social interaction patterns to extract representations of latent user preferences driving social behaviour. In practice, similarity scores between preferences are used to predict recurring interactions between users and their alters. To account for different behavioural patterns based on the cues and texture of different social activities, recommendations are calculated per context. The required communication protocol between HELIOS devices for exchanging parameters upon social interactions (which in the context of the GCS manager are effectively messages) is overviewed in Figure 4. The GCS module integrates the parameter exchanging protocol of Figure 4 to the message manager of Subsection 2.5. In total, there are three types of SocialGraphMiners provided by the social graph mining module, all of which run in the GCS module. These implement decentralized variations of promising graph mining algorithms investigated with a centralized analysis ^9^. Miner data (e.g. latent representations) are attached to the contextual ego network module’s data structures and saved through the latter. This way, any machine learning progress (for example that was reached after many social interactions) is not lost when closing and opening HApps. The three types of SocialGraphMiners are the following:

**RepeatAndReplyMiner.** Performs interaction recommendation based on chronological order. That is, the last interacted users are re-recommended. This is a common functionality of messaging platforms that helps users immediately reply to incoming messages. It does not send any data.

**Graph Neural Network Miner (GNNMiner).** Performs interaction recommendation based on latent interaction preference matching. To do this, it maintains an in-device estimation of ego and alter interaction preferences, as perceived by the user’s devices. Preferences are computed per context so that the ego and recently interacted alters in that context are assigned similar preferences but also preserve to an extent the preferences received by fragments of the miner running in alter devices. Even if preferences are updated asynchronously and only when other messages are sent, this scheme eventually converges to all devices forming similar representations about users in the same contexts of their contextual ego networks.

**Personalized PageRank Miner (PPRMiner).** Improves pretrained machine learning algorithms that classify device users (e.g. as the content-aware profiling does) by diffusing their outcomes through the heterogeneous social graph ^10^, for example through the stochastic equivalent ^11^ of random walk with restart across graph nodes. Using this miner to propagate predictions sets up a decentralized implementation of decoupled graph neural networks architectures that uses the machine learning algorithm as its base and performs diffusion to improve its outcome ^12^.

If multiple miners are defined (depending on HApp needs, there can be multiple instances of each miner running in parallel, for example bearing different machine learning hyperparameters), these can be gathered either into a common interface that can switch between them or into a variation of that interface that aggregates their outcomes. The aggregation is performed through a geometric mean, so as to prevent different scaling factors and non-linearities of individual miners from biasing in favor of any one’s results. Besides enabling or disabling outgoing miner information, miner contributions to the combined score can also be enabled, disabled or exponentiated with a non-negative factor representing their importance (importance set to 1 when enabling miner contributions and 0 when disabling them). HApp users can be provided with the option to control which miners contribute to the aggregation, so as to better customize their experience.

***2.9.2 Content-aware profiling integration*.** The content-aware profiling module analyses local collections of images to understand user preferences. It provides two types of preferences; coarse and fine interest profiles that include a broad selection of potential user interests, to which the content-aware profiling module estimates scores indicating how much device users are interested. Fine interest profiles provide a more granular understanding of subtopics, whereas coarse interest profiles pertain to broader interest categories (e.g. sports, food, art). The GCS module wraps the functionality of the content-aware profiling module by initiating on-demand running. This performs incremental parsing of images that does not revisit previously-analysed ones. For estimated profiles to persist even when HApps are closed and reopened, we used the serialization and serialization functionalities of the contextual ego network module to store interest profiles (these are not sent to alter devices). The GCS module separates computationally intensive profile loading costs from deserialization of context networks, so that HApps are not encumbered with serial operations that would slow boot up and response times due to periodically saving contexts.

## 3 helios.TALK

helios.TALK (see *Software availability* ^13^) is a communication app built on top of the GCS and offers the opportunity to the users to engage with other people through efficient and feature-rich group communications. helios.TALK can be leveraged to engage with new people around a certain topic, and connect with people with common interests such as students, co-workers, singles, etc. This section presents the architecture and the functionalities of the release version of the helios.TALK application.

### 3.1 Implementation

**Figure 5.**
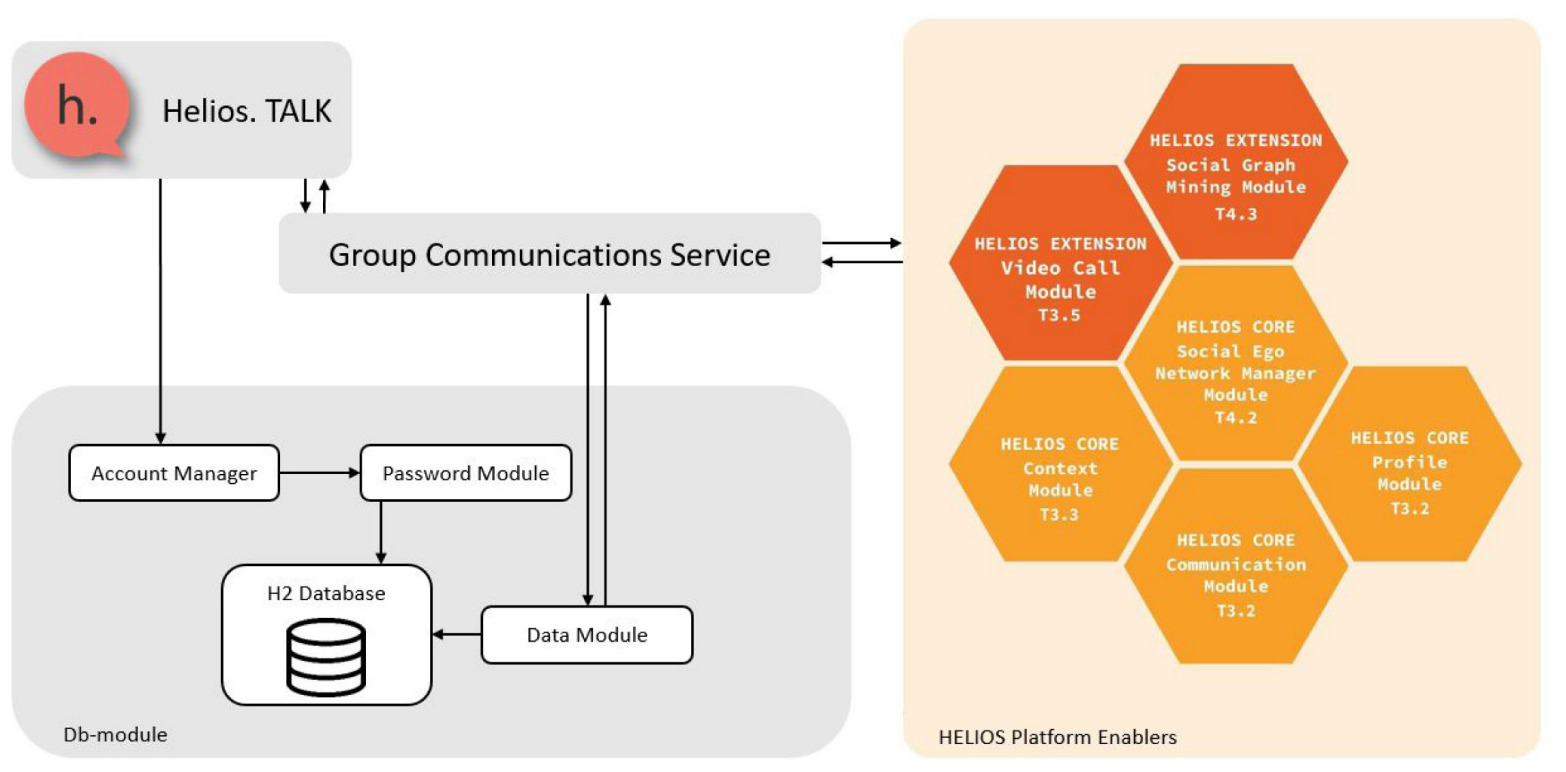
System design of helios. TALK.

***3.1.1 Architecture*.** helios.TALK follows a typical app architecture that consists of the *UI* layer, the *data* layer and the GCS that serves as a middleware between helios.TALK, the underlying database, and the integrated HELIOS core and extension modules. The selected type of system design supports the adoption of fundamental system properties, such as usability, maintainability, scalability, and extensibility. Figure 5 depicts the described system design.

**Database.** HELIOS is a peer-to-peer social media platform, meaning that no central authority stores users’ data. Thus, all data exchanged between peers are stored locally, in the user’s device. We use the embedded mode of H2 database, a relational database management system written in Java, to store data related to the user’s communications. The database is stored in encrypted files in the internal memory of the user’s phone, using the Advanced Encryption Standard algorithm.

**Account manager.** The account manager is responsible for validating the existing user account or creating a new one if it does not exist. Crypto module facilitates the account manager by estimating password’s strength, in the process of creating a new account, and storing this password encrypted in a file in the internal memory. When logging in, the user is required to enter their password and only if this password matches to the one given during setup then their can login to the application and have access to all previous communications.

**Data module.** The data module enables encoding and parsing data from/to bytes array to/from specific data types.

The GCS module enable the helios.TALK to provide several worth noting features to the users. To begin with, the P2P communication constitutes an integral part of the helios.TALK, which in combination with other privacy related features offers a safe way of communication. Briefly, the privacy oriented features are the following: authentication; encrypted one-on-one messages; receiving messages only from friends. Moreover, helios.TALK takes advantage of a data structure related to user’s Social Graph that offers functionalities about its creation (nodes, edges), management, and storage. The social graph represents the personal social network of a user. The nodes represent the alters added in a specific context, and the edges represent the relationships. In addition, users can create location and spatiotemporal contexts, keep track of their state (i.e., active/inactive), and share them with their friends. Other than that, helios.TALK supports different types of groups and enable users to handle them properly. It is noteworthy that by leveraging the provided algorithms, helios.TALK provides next interaction recommendations, content discovery functionality, and interest profiles based on the collection of images to the users. Finally, users can create their own profiles for each context, share their contacts, send text/image/file messages, and perform Video Call with other users.

**Figure 6.**
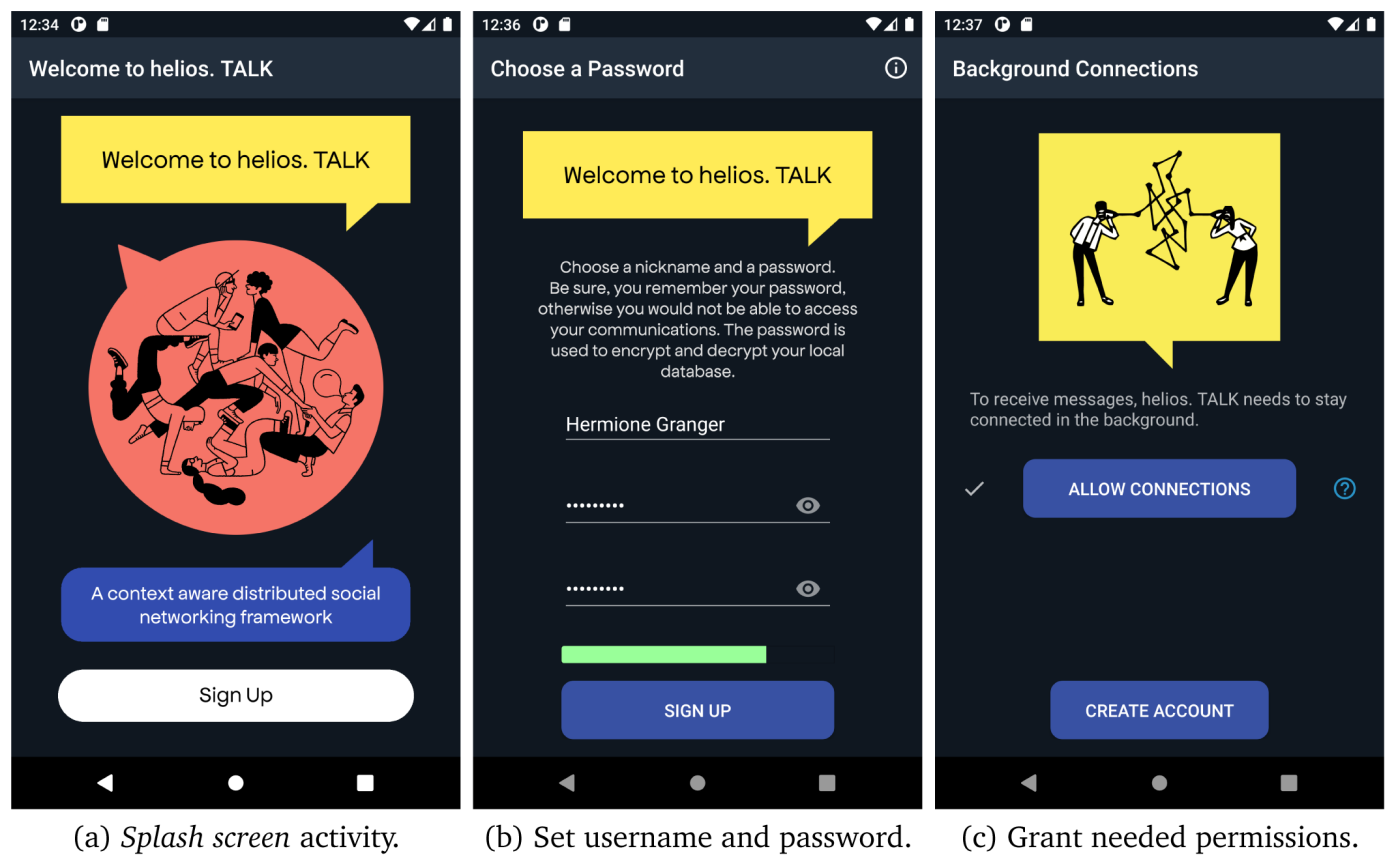
User registration steps.

***3.1.2 Use cases*.** The first time the users open the application, they are required to create an account by providing a username and a strong enough password that is used to open and decrypt the database contents. All user’s details are stored locally on a device’s database, as described in Subsection 3.1.1. In Figure 6, we present the different screens for creating a new account in helios.TALK.

After registration, users land into the *main* activity, where they can browse between three different tabs:

**Contacts.** Users are able to see their contacts and invite contacts to join a context.

**Chats.** They can access different active one-on-one or group conversations or can initiate a new conversation by creating a new private group/forum or interact directly with a specific contact.

**Fav(s).** Users can have quick access and navigate on messages received in one-on-one or group messages that they have marked them as “favourite” during conversations. This allows users to archive useful conversation points to go through later.

**Figure 7.**
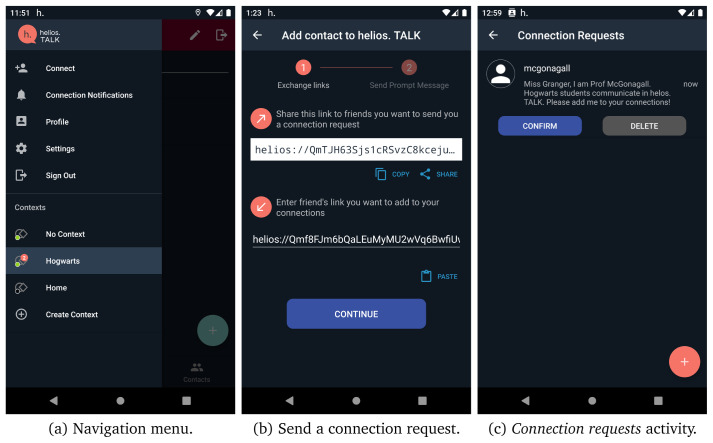
Steps to add a contact.

However, as the user has not added any contact, these tabs do not contain valuable data yet. By tapping on the navigation menu (i.e., top-left button), which is presented in Figure 7 (a), the user can navigate though the most helios.TALK functionalities. The following menu items are available:

**Connect.** The users are able to manage the pending connection requests or add new connections in their contacts by sharing their HELIOS identifier with other friends in different platforms and waiting for their friend to send a connection request. Otherwise, if a friend already shared their HELIOS identifier, then they can proceed by sending a connection invite, by giving them a nickname and sending a small message along with the invitation. Figure 7 presents the different screens in helios.TALK for sending a connection request and accepting/rejecting a connection request, a peer has sent you.

**Connection notifications.** Users can handle (accept/reject/remove) the pending context/group/forum invitations.

**Profile.** Users can modify their profile information, such as profile image, gender, country, work, university, interests, and personal quote.

**Settings.** Users can adjust display, security, notification, and privacy settings according to their preferences.

**Sign out.** Users sign out and leave the application.

**Contexts.** Users can navigate through their contexts by selecting one or create a new context by tapping on create context option. 

**Figure 8.**
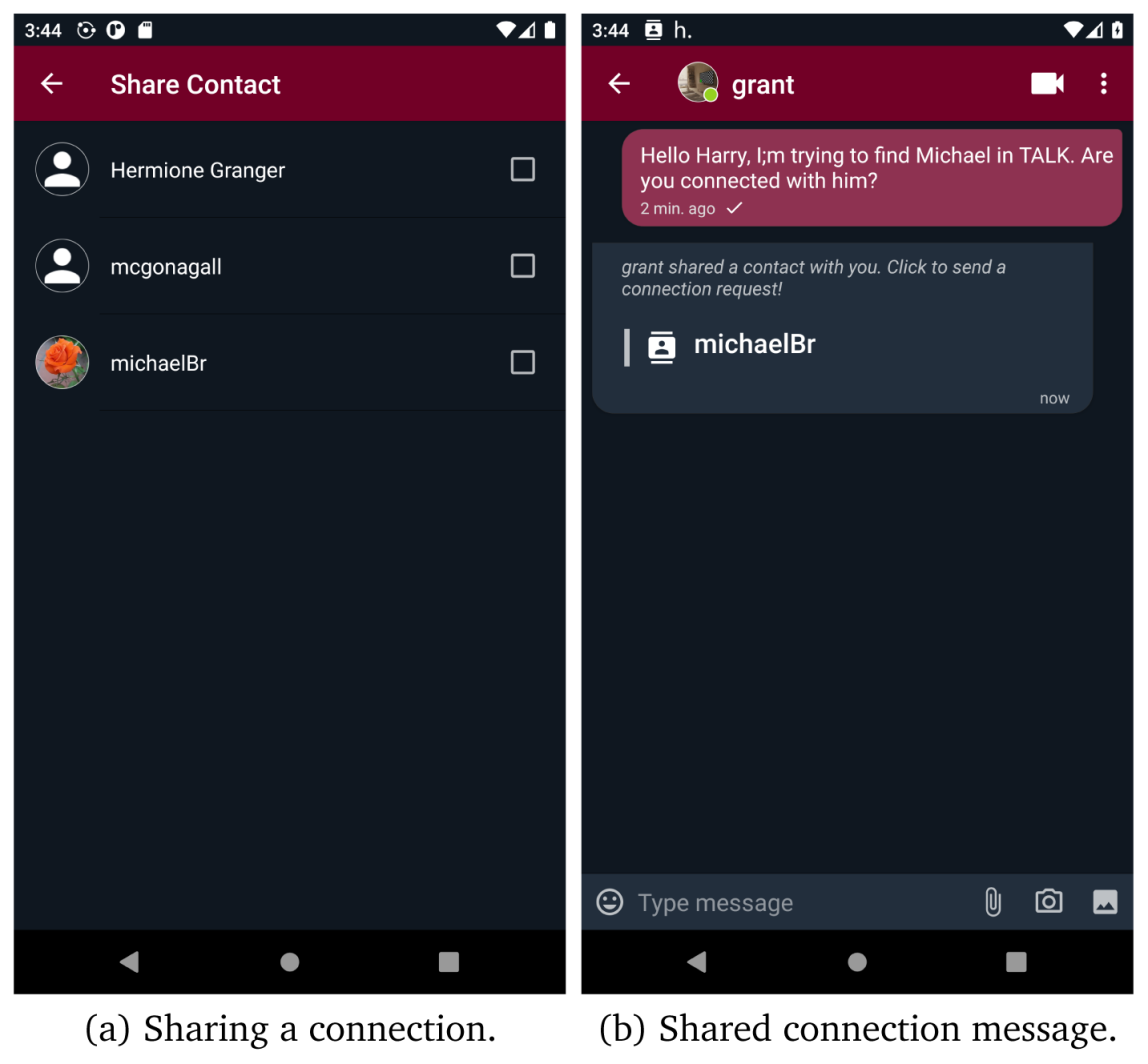
Share contacts screens.

Moreover, if a new connection request, invitation or message is received, an indicator is added to *connect*, *connection notifications* or a certain context, respectively. Users can also share their connections, thus simplifying the connection procedure. Users who receive a shared connection message, can send a connection request by tapping on that message. Figure 8 presents the exploitation of the share connection functionality to add a new connection.

**Figure 9.**
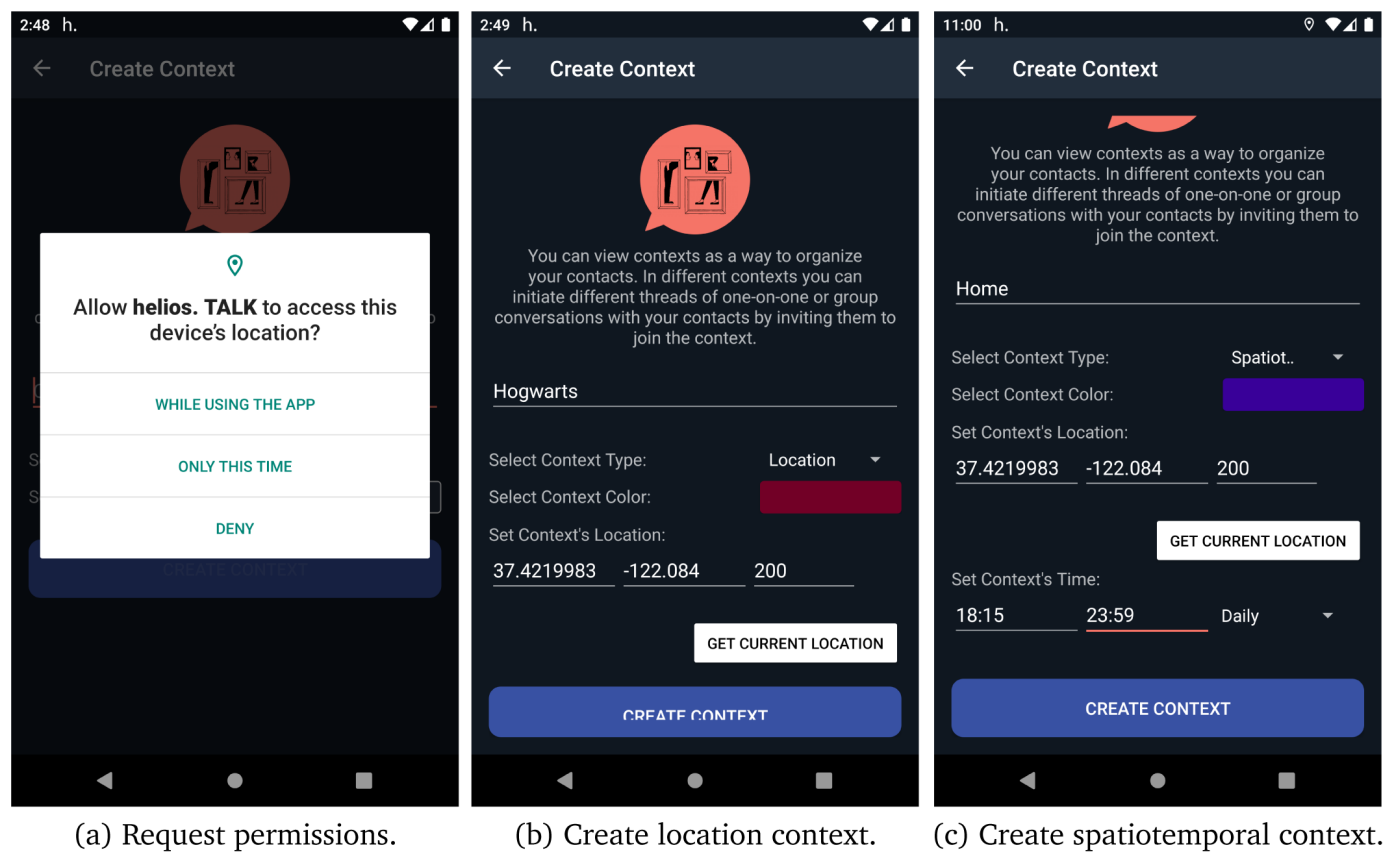
Steps to create a context.

As we have already discussed, one of the main characteristics of HELIOS platform is the notion of contextuality. Users can interact in different contexts and form different relationships. After registration, the GCS automatically registers the user to their first default context named “No Context”. All user’s new connections are automatically added in that default context. If a user taps on menu item create context, as depicted in Figure 7 (a), the *create context* activity opens. In the *create context* activity, as presented in Figure 9 (b) and (c), users can define a name and a colour for the context they want to create and they can also link a context to a specific location and a radius in meters (the default is 200 *m*). The application ask user to give access to location in order to be able to link contexts with specific locations. Additionally, users can create spatiotemporal contexts by adding both location and time information to the context, as it depicted in Figure 9(c). After creating a context, users are able to share the context with their contacts and initiate different threads of conversation in different contexts, one-on-one or group. In *invite contacts to context* activity users can share the context with their connection, if not already invited.

As contexts can be active or inactive, as described in Section 2.7, the corresponding state indicators are visible in the navigation menu, as presented in Figure 7 (a). Moreover, helios.TALK listens for context’s state changes and updates the UI (user interface) by setting as selected context the latest activated one. It should be noted that the selected context keeps updated, even if the application runs in background.

**Figure 10.**
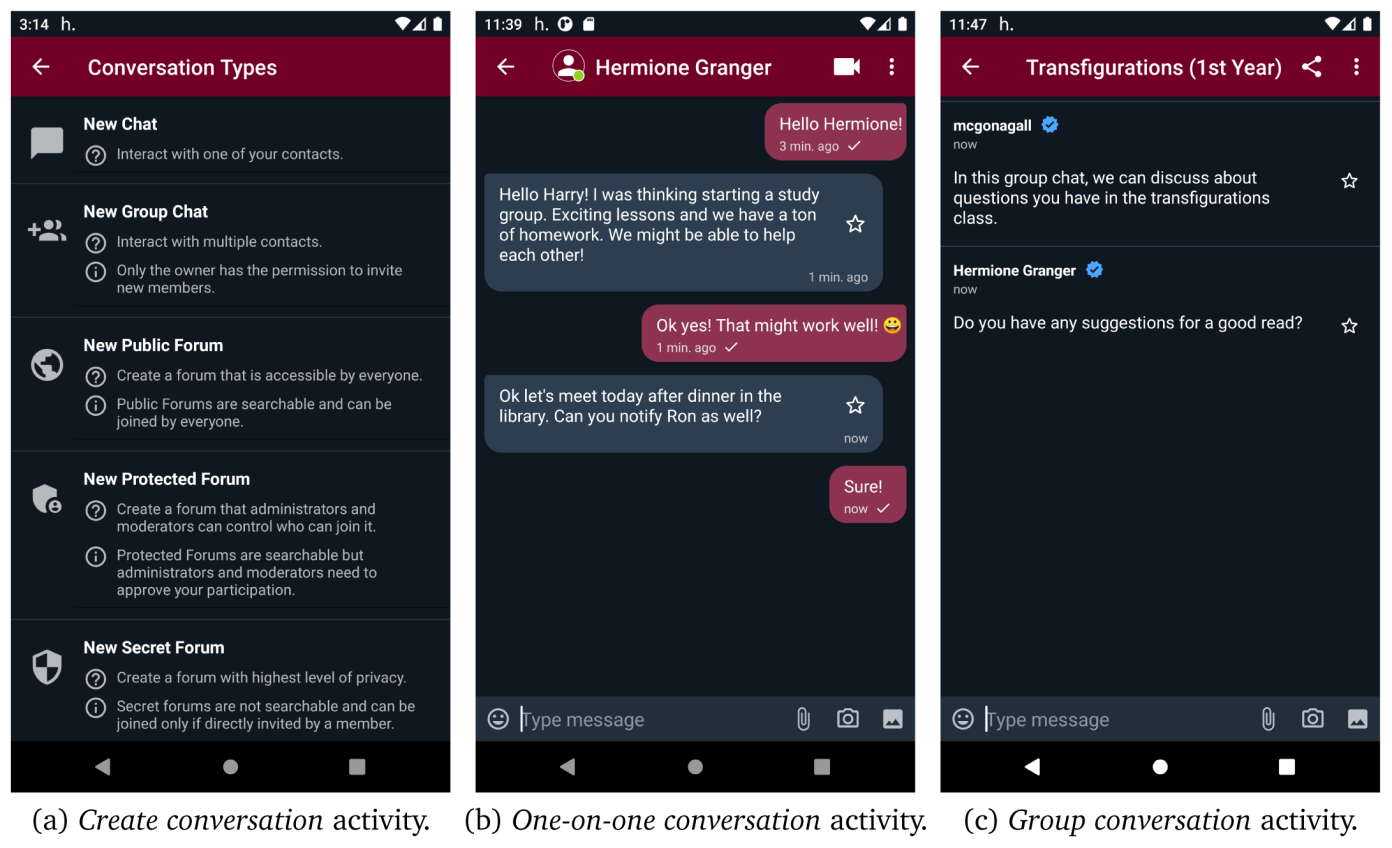
User interface for creating new conversations.

**Figure 11.**
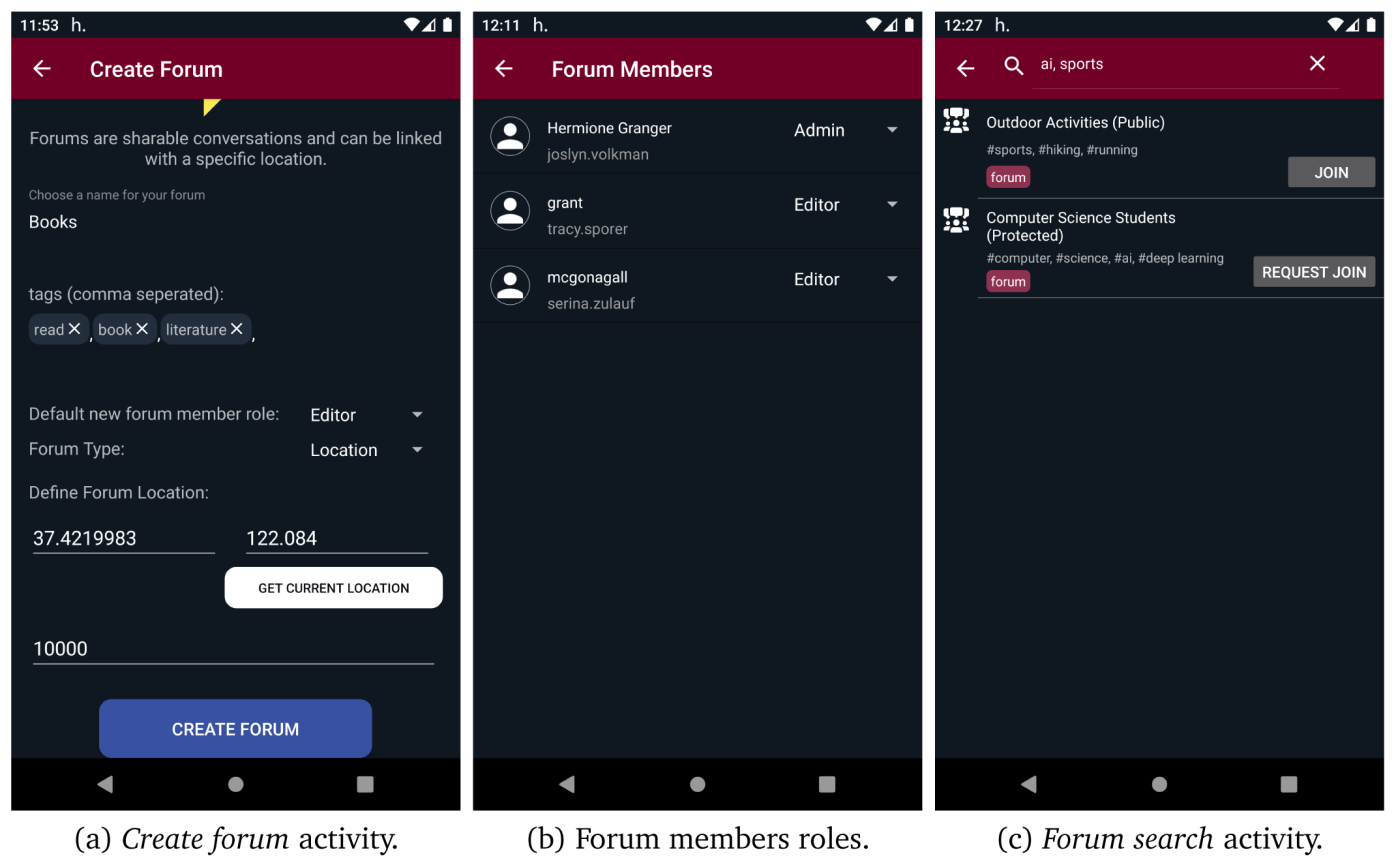
Forum-related user interfaces.

**Figure 12.**
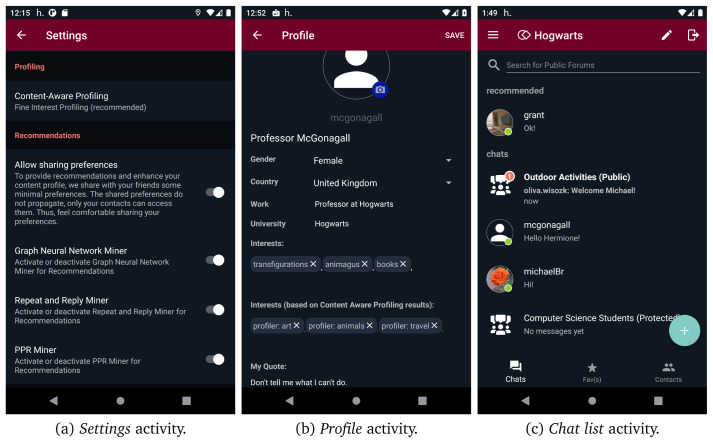
Activities related to data mining, profiling and recommendations in helios. TALK.

Figure 10 (a) demonstrates the *create conversation* activity of helios.TALK where users can opt for creating a new conversation (i.e., one-on-one chat, group chat or forum) to interact with other peers. If a user opts for creating a **new chat** (i.e., one-on-one-conversation), the *contacts* activity opens in order to choose the contact they want to start chatting with. In Figure 10 (b), the UI of the one-on-one conversations is presented. If more than two users want to interact with each other, they can create a group chat through the new group chat option. Moreover, as presented in Figure 11 (a), the options new public/protected/secret forum direct the users to the *create forum* activity, from where they can give a name, some tags, the default new members role, and connect the new forum with a certain location by giving geo-coordinates. If the new forum is placed in a location or spatiotemporal context, its geo-coordinates should be in the range of context’s location. Both forums and groups share the same conversation UI, as depicted in Figure 10 (c). In forums, administrators and moderators have access to the member list and can modify member roles, as depicted in Figure 11 (b). Moreover, public and protected forums can be discovered through search functionality. As it can be seen in Figure 11 (c), users are able to search for public/protected forums and join or request to join any public or protected forum found through search functionality, respectively. However, they cannot discover or join secret forums as they are not searchable and can only be joined via direct invitations. Additionally, users can access their profile and edit it from the navigation menu. They can define different profiles in different contexts, even use a different alias, define different interests etc. Apart from the interests that are defined by the user, the interests provided by content aware profiling are also presented. Figure 12 (b) illustrates the *profile* activity screen.

After users have been connected with at least three peers, one or more interaction recommendations provided by social graph mining are available in *conversation* activity, as shown in Figure 12 (c). Furthermore, through *settings*, users can adjust both their content aware profiling and mining preferences. They can opt for the profiler type (i.e., *coarse* or *fine* profiler) or disable this functionality. Moreover, they can enable/disable each one of the three available miners (i.e., graph neural network miner, repeat and reply miner, and PPR miner). Figure 12 (a) presents the UI of mining and content aware profiling preferences. Finally, helios.TALK employs an intent mechanism that enables users to share external content (i.e., text, images, videos) with their connections through *share content* activity. After the *share content* activity has started, users can choose the context and the contacts they want to share their content with.

### 3.2 Operation

The helios.TALK is an android application freely available on the Google Play Store and it can be installed on both smartphones and tablets with operation system Android 9 or newer. In addition, internet and location access are required to enjoy fundamental helios.TALK features, such as communication through different networks and location contexts or forums.

### 3.3 Discussion

There already exist social networking applications, some of the most popular being Briar, WhatsApp, Facebook Messenger, and Slack. However, these find it difficult to strike a balance between privacy-aware decentralization and user-friendly features. A comparison of theirs and helios.TALK’s available features is presented in Table 7. We can see that, the capabilities provided by the GCS module and our design considerations have generated a feature-rich app.

**Table 7.** Comparison between helios. TALK and other popular communication applications.

Feature	helios.TALK	Briar	WhatsApp	FB Messenger	Slack
decentralized	✓	✓			
image, video, voice, document messages	✓	only images	✓	✓	✓
voice calls	✓		✓	✓	✓
voice conferencing			✓	✓	✓
E2EE	✓	✓	✓	✓	
E2EE by default	✓	✓	✓		
encryption in transit	✓	✓	✓	✓	✓
channels/contexts	✓				✓
search messages			✓	✓	✓
search public forums	✓				✓
search for forums based on location	✓				
delete messages from receiver				✓	✓
user mentions			✓	✓	✓
emojis, stickers, GIFs	only emojis		✓	✓	✓
reactions on messages				✓	✓
no-name policy	✓				
multiple identities	✓				
private forums	✓	✓			✓
protected forums	✓				
public forums	✓				✓
location-based and spatiotemporal forums	✓				
blogs		✓			
day status (insta-stories like feature)			✓	✓	
chat themes	✓		✓	✓	
next interaction recommendations	✓				
contact introductions		✓			
user profiling (content-aware profiling)	✓				
**Number of features**	20	8	11	12	13

In particular, the introduced app allow users to build meaningful relationships in different contexts. Users can link contexts with specific locations or time spans and contexts can be activated when the location or time criteria are met. Even though search in centralized platforms can be considered a standard feature, in decentralized platforms it is a very challenging task. Briar, which is also a peer-to-peer messaging application, does not allow users to search for public forums. The helios.TALK resource discovery mechanism allow users to search for public forums also based on location to discover public discussions that are happening nearby. Support for multiple identities is another feature of helios.TALK, not met in other communication app since the application allows users to define different profiles/identities in different contexts. helios.TALK offer a variety of additional features to make user experience more playful and engaging. Mining user’s data to offer meaningful recommendations is a task usually implemented using centralized servers. Next interaction recommendations, as well as content-aware profiling are some features built on top of such mining tasks and even though FB Messenger provides some of these features most of them become available through the Facebook app and not directly through FB Messenger.

The thorough documentation of the features we meet in different communication applications gives us a better understanding of the purpose of each application. In short, although there is an abundance of messaging applications in the digital communications industry, what we are trying to achieve is to provide a secure messaging application that does not sacrifice the wealth of features and the overall user experience, which is technically challenging to implement in decentralised platforms. We believe such features are driving users to centralised platforms, even though they are aware they are sacrificing their privacy. What we are aspiring to offer to users is a smooth and feature-rich user experience that does not pose any risks to their personal data.

## 4 Conclusions

In this paper we introduce the helios.TALK and the GCS module developed in the scope of the HELIOS platform. GCS leverages most of the HELIOS modules to incorporate features, such as resource discovery, file sharing, user profiling, content availability, community detection and next interaction recommendations. helios.TALK constitutes a decentralised messaging application that leverages the HELIOS GCS module to allow users to connect and start a conversation, one-on-one or in groups in different contexts in a user friendly environment. Most of the decentralized communication applications focus on providing security and privacy features to ensure secure communications but they lack additional features that provide an enriched and fun communication experience to users. Our aspiration is to offer to users a rich group messaging experience, while ensuring their personal data is secure and not shared with any platform or application provider.

## Data availability

No data are associated with this article.

## Software availability

GCS module source code available from: https://github.com/helios-h2020/h.extension-groupcommunications

Archived source code at time of publication: https://doi.org/10.5281/zenodo.5912923 ^2^

License: GPL-3.0

helios.TALK software available from: https://play.google.com/store/apps/details?id=eu.h2020.helios_ social.helios.talk

helios.TALK source code available from: https://github.com/helios-h2020/h.app-Helios-Talk

Archived source code at time of publication: https://doi.org/10.5281/zenodo.5912921 ^13^

License: GPL-3.0

## Ethics and consent

Ethical approval and consent were not required.

## Footnotes

^1^The messaging module implements this with libp2p’s topic subscription mechanism, where topics correspond to the GCS module’s groups.

^2^Factory is a design pattern in Java used to abstract the instantiation of objects, such as pending contacts, through their properties that automatically determine called constructors.

^3^Modern neural methods can understand more expressive queries, such as “what is the capital of Germany?”, and provide targeted answers. These methods are currently undergoing intense research and have been deployed at some big search engines but their integration is still limited.
