# Supplementary material for: Helios.TALK: A decentralised messaging framework that preserves the privacy of users
Source: Open Res Eur. 2023 Oct 18;2:29. Originally published 2022 Mar 4. [Version 2] doi: 10.12688/openreseurope.14421.2 (PMC11809478; doi:10.12688/openreseurope.14421.2)
Supplement: Peer review history

# Reviewer report: Reviewer response for version 2

**DOI:** 10.21956/openreseurope.18026.r47929

**Published:** 2024-12-28

**Authors:** Ana Filomena Curralo

**Affiliations:** 1 Instituto Politecnico de Viana do Castelo, Viana do Castelo, Viana do Castelo District, Portugal

The article introduces Helios.TALK, a decentralized messaging tool built on the HELIOS platform, designed to address privacy concerns in centralized messaging apps. Utilizing the Group Communication Service (GCS) module, it enables peer-to-peer communication, end-to-end encryption (E2EE), context-aware interactions, and group management.

 The authors emphasize the advantages of decentralization in fostering user trust and data security, comparing Helios.TALK to platforms such as WhatsApp, Briar, and Slack. Key features include spatiotemporal contexts, user profiling, and next-interaction recommendations, all within a decentralized framework. However, the article also addresses challenges, including limited adoption and fewer features compared to centralized alternatives.

 While the article presents a well-conceived and technically sound framework for decentralized messaging, the rationale behind Helios.TALK's development could be better substantiated with empirical data and user feedback. Addressing these critical points would significantly strengthen the scientific rigor of the article, enhancing both its impact and clarity.

 The technical architecture is thoroughly outlined, detailing modules such as the GCS, communication manager, and social graph mining. Key design considerations, such as scalability, modularity, and encryption protocols, are well-supported by clear and detailed explanations.

 The description would benefit from additional information on stress-testing the tool under heavy usage or in edge-case scenarios, such as network failures or large group sizes. Furthermore, including performance benchmarks that compare Helios.TALK to both centralized tools like WhatsApp and decentralized alternatives like Briar would provide a more comprehensive evaluation of its capabilities.

 The article effectively identifies key issues with centralized messaging platforms, such as data breaches and the lack of user control, and advocates for decentralization as a viable solution to improve privacy and trust. The HELIOS platform is introduced with a focus on decentralized communication through modular components and privacy-by-design principles. However, the discussion lacks concrete examples and real-world evidence, such as case studies of privacy violations in centralized platforms.

 Additionally, the article offers limited comparison with existing decentralized tools like Briar or Telegram, and does not incorporate user feedback or address the unique challenges of decentralized systems. To strengthen its rationale, the article should include real-world examples, benchmark comparisons, user feedback, and empirical performance data, especially focusing on scalability and edge-case testing. These improvements would make the arguments more compelling and scientifically robust.

 Although the conclusions align with the article's objectives, they are not sufficiently supported by empirical data or comparative analysis. A more detailed assessment of performance and user experience is needed for a stronger, evidence-based conclusion.

 While the conclusions are consistent with the feature descriptions and design goals, they lack empirical support for the performance claims. Comparisons with other tools are primarily conceptual, and edge cases are not addressed. To bolster the conclusions, the paper should include quantitative performance testing, comparative analysis, user studies, and edge-case testing. These additions would provide measurable evidence to substantiate the claims and conclusions.

Is the rationale for developing the new software tool clearly explained?

Partly

Is the description of the software tool technically sound?

Yes

Are sufficient details of the code, methods and analysis (if applicable) provided to allow replication of the software development and its use by others?

Yes

Is sufficient information provided to allow interpretation of the expected output datasets and any results generated using the tool?

Yes

Reviewer Expertise:

Digital Design:UI/UX Design Systems;Interaction Design and Prototype; Rensposive Design; Usability text; Human-Computer Interaction (HCI)                                                 Product Design:Design Thinking and Innovation; Emotional and Sensory Design; Smart Products and IoT (Internet of Things) and Design for Health and Well-being.

I confirm that I have read this submission and believe that I have an appropriate level of expertise to confirm that it is of an acceptable scientific standard.

# Reviewer report: Reviewer response for version 2

**DOI:** 10.21956/openreseurope.18026.r36677

**Published:** 2024-01-08

**Authors:** Hà Xuân Sơn

**Affiliations:** 1 RMIT University, Ho Chi Minh City, Vietnam

The paper introduces a decentralized messaging framework called HELIOS Group Communication Services (GCS) and a GCS-based Android application. The two tools aim to provide a secure yet fully functional alternative to traditional social media platforms.

 Strengths and reasons to accept:

 + The paper provides a detailed and easy-to-read explanation of the tools' purposes and functions. The authors clearly explained how the proposed project can address the mentioned problems.

 + The GCS offers a set of functions expected from a messaging platform, such as messaging, user management, and searching. 

 + The paper tackles several challenging problems for a communication framework, such as a context manager and social graph mining integration.

 + The authors introduced Helios.TALK, a working Android app, to illustrate the capabilities of GCS.

 Weaknesses

 + The authors have stated in the introduction that despite the efforts made, there are still significant risks associated with centralized models of online social networks. This has resulted in the growth of alternative Decentralized Online Social Networks (DOSNs). I would like to understand better the risks associated with centralized models and how the traditional approaches have been insufficient in addressing them.

 + Currently, there is only one comparison with Briar regarding the number of functions. There should be a more comprehensive comparison with existing projects. I would also like to compare the features' stability and quality. For example, does GCS have less latency?

 + As the project also wants to improve user experience, I recommend a survey to see how users feel about the application's usability.

Is the rationale for developing the new software tool clearly explained?

Partly

Is the description of the software tool technically sound?

Yes

Are sufficient details of the code, methods and analysis (if applicable) provided to allow replication of the software development and its use by others?

Yes

Is sufficient information provided to allow interpretation of the expected output datasets and any results generated using the tool?

Yes

Reviewer Expertise:

security and privacy in Android apps

I confirm that I have read this submission and believe that I have an appropriate level of expertise to confirm that it is of an acceptable scientific standard, however I have significant reservations, as outlined above.

# Reviewer report: Reviewer response for version 2

**DOI:** 10.21956/openreseurope.18026.r36674

**Published:** 2024-01-02

**Authors:** Andrea Michienzi

**Affiliations:** 1 Department of Computer Science, University of Pisa, Pisa, Italy

In this paper, the authors present the Group Communications Service module of the HELIOS platform that can be used to support different types of group conversations. The paper contains an excellent description of the module and of the Helios.TALK application implemented using the module. Here are the aspects where I think the authors could improve in their paper:

 - The main motivation behind proposing a decentralised messaging app is unclear. I recommend the authors elaborate more in the introduction section about this aspect, in particular on what concerns the progressive decentralisation of online social services (Diaspora and Mastodon are two important platforms in this regard), and why decentralisation can be beneficial also in the scenario of messaging apps.

 - The social media platform HELIOS is introduced abruptly in the paper. I recommend that the authors smooth the passage, by introducing what HELIOS is doing differently with respect to other analogous platforms.

 - To make the paper more self-contained, I recommend the authors either discuss more details about the HELIOS platform or provide sufficient pointers to existing literature.

 - In Table 5, "Free join" and "Request join" seem mutually exclusive options, therefore the authors should consider merging the two columns

 - The authors state that the mining manager "could be of interest to both users and privacy-respecting personalized advertisement, such as monetized HApps that periodically exchange collections of advertisements and display only the ones that best match user interests" which I think goes in contrast with respect to the original purpose of HELIOS. I recommend the authors clarify this aspect.

 - The conclusion does not point to any possible future work.

 - Lastly, I recommend the authors include more references to scientific papers, to give the readers more pointers in case they seek to delve into the various topics touched by the paper.

Is the rationale for developing the new software tool clearly explained?

Partly

Is the description of the software tool technically sound?

Partly

Are sufficient details of the code, methods and analysis (if applicable) provided to allow replication of the software development and its use by others?

Yes

Is sufficient information provided to allow interpretation of the expected output datasets and any results generated using the tool?

Yes

Reviewer Expertise:

Online Social Netowrks, Blockchain

I confirm that I have read this submission and believe that I have an appropriate level of expertise to confirm that it is of an acceptable scientific standard, however I have significant reservations, as outlined above.

# Reviewer report: Reviewer response for version 2

**DOI:** 10.21956/openreseurope.18026.r36680

**Published:** 2024-01-02

**Authors:** Kalman Graffi

**Affiliations:** 1 Technical University of Applied Sciences Bingen, Berlinstraße, Germany

The article proposes a p2p-based architecture for one-to-one and group communication. It builds on a technical network representation of the ego network of the participants (see works of Guidi et al) and describes a est of modules building the architecture to implement chat and (public, private, invitation-based) forums.

 The evaluation is based on a feature comparison with a selected set of alternative tools of similar functionality.

 The main question is:

 - Are the design decisions sufficiently described and discussed?

 - Is the technical depth of the architecture description sufficiently detailed for the interested reader to learn from it?

 - Is related work sufficiently considered?

 - Is the quality of the proposed approach sufficiently (measured and) discussed?

 The reviewer aims to clarify these questions in order to allow an assessment of the article.

 The authors propose to use a p2p overlay network based on the ego network of the user. User information "Comprises basic user information (identifier, alias, and profile picture) and a text message." Further the authors write "Messaging is the main functionality of the GCS module. Thus, a number of processes are developed to facilitate one-on-one and multiway communication." As a last quote in this context: "To begin with, the P2P communication constitutes an integral part of the helios.TALK, which in combination with other privacy related features offers a safe way of communication. Briefly, the privacy oriented features are the following: authentication; encrypted one-on-one messages; receiving messages only from friends. "

 hus correct identities are essential to allow for authenticated and secure (integer, confidential) communication in the ego network.

 With regard to the network quality, the question arises how users can bootstrap their participation in the network? In an ego network, as well as stated by the authors, messages are only received from friends. How is a user joining the network, with initially 0 friends or friends that are offline? How can users interact with the system when no fried in offline or in case that the user does not want to expose friendships and only wants to browse public forums?

 The authors should explicitly describe the entry process for new users and the modus operandi for users who do not want to have "friends" in the system.

 As a followup on identities, the question arises how "friends" are identified. How is a friend request authenticated and linked to a real identity of a friend in contrast to someone claiming to be that person?

 When a user creates a new account, the authors describe in the crypto module that the password of the user is stored encrypted in the device's internal memory. That is fine (although it limits the portability and changing to another device), a further aspect is mentioned with regard to sending conversation messages:

 "For security purposes, one-on-one messages are also encrypted with the Advanced Encryption Standard (AES) protocol and the AES key is encrypted with the Rivest–Shamir–Adleman (RSA) protocol using the security & privacy module"

 At what time is an RSA key pair generated and how are the public keys of friends verified? Can anyone claim to be a specific friend?

 The authors should summarize the security related elements and properties of their system. Currently it is unclear how users are authenticated, and later with regard to the forum entries, whether forum messages are integer and authenticated. The reviewer will elaborate on this point later in the review.

 Picking up the discussion on the technical properties of the p2p overlay. We learn that "Moreover, helios.TALK takes advantage of a data structure related to user’s Social Graph that offers functionalities about its creation (nodes, edges), management, and storage. The social graph represents the personal social network of a user. "

 How is the current IP address of a user identified by this friends? P2P networks typically suffer under network churn, i.e. users going offline and online. Especially for apps on smartphones it might happen that IP addresses change frequently, depending on whether cellular or Wifi connectivity is used. Some celluar network ISPs strictly forbid p2p communication in their cellular networks. How does the network cope with churn?

 Further we learn that all data is stored in local SQL databases (on each peer). The authors should add a discussion on the quality properties of this storage. In specific, whether the data storage provides availability and security. What happens if a users that is currently offline submits a message to a friend or in a forum. Obviously when the user is currently offline, it will not be sent. Will that message be sent if the user comes online again? What if 10 users sent messages to a forum, how will the order be determined? How long can messages be "inserted" or "retransmitted" before they get obsolete? Can a user go offline for 1 year, write 1000 messages in that year and expect that his messages are sent and "sorted in" correctly once online again?

 Do the users have a local view only or is there and attempt to synchronize the various local SQL storages?

 The overlay network further proposes a search functionality described in the ressource discovery module: "queries are relayed across the connected users in the heterogeneous social graph, a function that is known in the literature as unstructured peer-to-peer search." This is regular flooding that had lead to the collapse of some Gnutella networks. Thus it is essential to limit the range of such a flood. What parameters are chosen here and how is a common flood in circles prevented (as friends of friends are often also friends of us).

 This part is crucial and should be sufficiently elaborated and discussed by the authors, as it lead to the downfall of previous unstructured p2p networks.

 One reviewer addressed the question for scalability, to which the authors responded:

 "The developed modules do not enforce a particular communication framework; instead, they allow for integration with various decentralized communication protocols. Hence, we regard evaluating the scalability of the proposed framework as beyond the scope of this paper."

 As technical constraints are defined (communication only with friends, need for being online, search through flooding) that are implementation independent, the impact of these decisions should be evaluated.

 With regard to security:

 1. How are malicious nodes addressed that aim to spoof identities and messages or aim to potentially break data consistency across various nodes?

 2. A "Private Group ... is described by a unique identifier, password, context identifier, immutable name, and owner id. All members have read and write permissions by default" As all users share a common password to be able to read and write to the group, how is authentication (of the author) and integrity (not overwriting / deleting contents of others) guaranteed?

 3. Four roles exist in Forums, the same question applies here. Is authentication and integrity of the messages provided?

 4. The mining manager as well as the context-aware profiling manager seem quite invasive, observing social interactions and "analyzing local collections of images". The users should discuss the dangers of crawling through the images of a user on his phone for a functionality not related to the images of the user. It would be good to draw a line to trojan malware that sometimes behaves identically.

 Eventually, the authors should add a quantitative evaluation to the article addressing the concerns with regard to the scalability of the system as well as depicting the traffic and storage costs. The current qualitative comparison of a handful of alternatives is not convincing as not every feature is equally important, such as the unclear value of "multiple identities", "chat themes", "location-based and spatiotemporal forums". Counting the features while ignoring potential drawbacks (scalability, availability, authentication of users) seems arbitrary.

 Further more, the authors only compare helios.talk to Briar as other decentralized approach, while in their own reference [4] (Masinde et al. -Peer-to-peer-based social networks: A comprehensive survey.) 23 (twenty-three) p2p based online social networks are discussed, out of which 8 have reached prototype status and 1 (Twister) being already deployed. 14 our of 23 propose social graph traversal, 6 out of 23 use an unstructred p2p network.

 As final point it needs to be mentioned that some of the references lead to nowhere, such as the link [2], [3], [6] and [7] and anything related to https://helios-h2020.eu

 In summary, compared to the technical description of some of these 23 other p2p-based OSNs, this article should be extended with more details on

 -the network properties (joining process, coping with no friends, coping with churn, scalability of the search functionality, managing identities on various devices, authenticating friends)

 - the data storage properties (consistency of (forum) data across various nodes, data authentication and integrity, coping with storage/message events on offline nodes)

 - the security properties (source of RSA keys, authentication of communication partners without public key infrastructure, protection against users overwriting content of other users with WRITE rights)

 - the evaluation by adding a quantitative evaluation of the performance and costs of the running system. With the apps being available, i.e. the system being functional, it should be doable to test various scenarios to quantify the operation times and traffic costs, the consistency of data, the impact of flooding.

Is the rationale for developing the new software tool clearly explained?

Yes

Is the description of the software tool technically sound?

No

Are sufficient details of the code, methods and analysis (if applicable) provided to allow replication of the software development and its use by others?

No

Is sufficient information provided to allow interpretation of the expected output datasets and any results generated using the tool?

No

Reviewer Expertise:

peer-to-peer networks, opportunistic networks, p2p-based online social networks, secure distributed systems

I confirm that I have read this submission and believe that I have an appropriate level of expertise to state that I do not consider it to be of an acceptable scientific standard, for reasons outlined above.

# Reviewer report: Reviewer response for version 2

**DOI:** 10.21956/openreseurope.18026.r36679

**Published:** 2023-12-22

**Authors:** Vincent Omollo Nyangaresi

**Affiliations:** 1 Tom Mboya University College, Homa Bay, Homa Bay County, Kenya

1) In the abstract, you need to state the existing decentralised messaging tools, point out their shortcomings and explain how your proposed tools address the identified issues.

 2) Towards the end of the abstract, provide some summary of the key findings based on the experimental outcomes.

 3) The entire manuscript should be properly cited and all the cited works should appear in the list of references.

 4) Towards the end of the 'Introduction', articulate the major contributions of this paper. This should be preferably in point form. After this, state how this paper is organised.

 5) You need to provide a pseudo-code to depict the operations of the proposed decentralised messaging tools.

 6) Include some future research scope in the 'Conclusion' section.

Is the rationale for developing the new software tool clearly explained?

Yes

Is the description of the software tool technically sound?

Yes

Are sufficient details of the code, methods and analysis (if applicable) provided to allow replication of the software development and its use by others?

Partly

Is sufficient information provided to allow interpretation of the expected output datasets and any results generated using the tool?

Partly

Reviewer Expertise:

IoT, cellular communications, network security, machine learning, TCP/IP, communication protocols

I confirm that I have read this submission and believe that I have an appropriate level of expertise to confirm that it is of an acceptable scientific standard, however I have significant reservations, as outlined above.

# Reviewer report: Reviewer response for version 1

**DOI:** 10.21956/openreseurope.15566.r30295

**Published:** 2022-11-03

**Authors:** George Drosatos

**Affiliations:** 1 Institute for Language and Speech Processing, Athena Research Center, Xanthi, Greece

This paper presents a framework for decentralized communication, namely Group Communication Services (GCS) that offers numerous functionalities, such as context-awareness and user profiling. In addition, an Android decentralized messaging application (i.e., Helios.TALK) that is built on top of the GCS is introduced. 

**Strengths and reasons to accept**:

- The GCS constitutes a useful open-source module that enables developers to create decentralized communication applications. 
- Helios.TALK is a well-designed decentralized messaging application published on Google Play Store.
- Both source codes are open source and available on GitHub under the GPL-3.0 license.
- The authors propose solutions for functionalities that are challenging to be applied in a decentralized manner (e.g., graph mining and user profiling).  
- The manuscript is overall well-written.

**Weaknesses and limitations**:

- The comparison of the proposed solution with other decentralized frameworks is incomplete. It would be useful to compare the Helios.TALK with apps that are built on top of the Ethereum Whisper.
- There is no mention of the scalability of the proposed solution. Does the increasing number of users affect the reliability of the application?

Is the rationale for developing the new software tool clearly explained?

Partly

Is the description of the software tool technically sound?

Yes

Are sufficient details of the code, methods and analysis (if applicable) provided to allow replication of the software development and its use by others?

Yes

Is sufficient information provided to allow interpretation of the expected output datasets and any results generated using the tool?

Partly

Reviewer Expertise:

Privacy-Enhancing Technologies, Information Security, Blockchain Technology, Biomedical Informatics, Data Mining, Social Media Analytics, and Machine Learning.

I confirm that I have read this submission and believe that I have an appropriate level of expertise to confirm that it is of an acceptable scientific standard, however I have significant reservations, as outlined above.

# Response

**Published:** 2023-09-28

**Authors:** Ioannis Sarridis

**Affiliations:** Centre for Research and Technology Hellas, Greece

We would like to thank the Reviewer for his valuable comments and suggestions. Since both concerns align with those raised by Reviewer 1, please refer to the respective responses in our reply to Reviewer 1.

# Reviewer report: Reviewer response for version 1

**DOI:** 10.21956/openreseurope.15566.r29864

**Published:** 2022-08-31

**Authors:** Qiben Yan

**Affiliations:** 1 Department of Computer Science and Engineering, Michigan State University, East Lansing, MI, USA

The paper proposes a decentralized messaging framework, called the HELIOS Group Communication Service (GCS), as well as an Android app built on this framework, called HELIOS.Talk. The framework is subsequently built upon the HELIOS peer-to-peer social media platform, which is not part of this paper. The authors elaborate on the design of the proposed framework and the app and compare their functionality with existing centralized mainstream messaging solutions.

 Strengths:

 + Decentralized messaging is an interesting, well-justified, and challenging problem.

 + Helios.TALK is a functioning Android app.

 + The paper is well-written.

 Weaknesses:

 - Unsupported claim

 - Insufficient comparison with existing solutions.

 - No performance and scalability evaluation.

 Detailed Comments:

 The idea of decentralized social networking is interesting, especially in the wake of multiple existing privacy and sociopolitical concerns. The proposed solution offers a decentralized social networking framework, which is both interesting and timely. Moreover, the framework operates upon a functioning distributed platform, and the proposed Helios.Talk app is available on Google Play Store and operates per specifications. The design of the solution is properly described and illustrated in the paper.

 However, this work has some major limitations that should be fixed.

 First, the authors claimed that **“**We believe such features are driving users to centralised platforms…” However, this claim is not proven. It’s unclear to me whether the features described in the paper are the key ones that decide the users’ interests in centralized platforms. More support is needed. 

 Second, Section 3.3 and Table 7 only compare Helios.Talk with the centralized solutions, such as WhatsApp, which is insufficient because there are proposed decentralized solutions, such as Ethereum Whisper that are both decentralized and claim similar functionality. ^1^ For example, what is the advantage of HELIOS HApps over other dApps? Why cannot a Web3 Whisper-based DApp be used instead of Helios.Talk?

 Third, the paper lacks a discussion and/or evaluation of the performance and scalability of the framework w.r.t. the user number increase. Specifically, how does the transaction throughput increase if the number of users grows N times? Are there any sensitive delays or storage limitations? These aspects are crucial for determining the practicality of the proposed solution.

 References:

 [1] EIP-627: Whisper Specification, 2017

Is the rationale for developing the new software tool clearly explained?

Partly

Is the description of the software tool technically sound?

Yes

Are sufficient details of the code, methods and analysis (if applicable) provided to allow replication of the software development and its use by others?

Partly

Is sufficient information provided to allow interpretation of the expected output datasets and any results generated using the tool?

Partly

Reviewer Expertise:

Cybersecurity, including blockchain system and security.

I confirm that I have read this submission and believe that I have an appropriate level of expertise to confirm that it is of an acceptable scientific standard, however I have significant reservations, as outlined above.

## References

[1] : EIP-627: Whisper Specification. *Ethereum Improvement Proposals* .2017; Reference source

# Response

**Published:** 2023-09-28

**Authors:** Ioannis Sarridis

**Affiliations:** Centre for Research and Technology Hellas, Greece

We would like to thank the reviewer for the thorough review. Kindly find our responses below.

 Comment 1: *First, the authors claimed that **“**We believe such features are driving users to centralised platforms…” However, this claim is not proven. It’s unclear to me whether the features described in the paper are the key ones that decide the users’ interests in centralized platforms. More support is needed.*

 Response 1: In response to this, we have incorporated a paragraph in the Discussion Section of the revised manuscript to provide further elaboration.

 Comment 2: *Second, Section 3.3 and Table 7 only compare Helios.Talk with the centralized solutions, such as WhatsApp, which is insufficient because there are proposed decentralized solutions, such as Ethereum Whisper that are both decentralized and claim similar functionality. ^1^ For example, what is the advantage of HELIOS HApps over other dApps? Why cannot a Web3 Whisper-based DApp be used instead of Helios.Talk?*

 Response 2:  We selected applications for comparison based on their comprehensive set of functionalities, which often led us to predominantly centralized solutions. However, to address the reviewer's valid point, we have now included a comparison with the decentralized app "Status" to offer a more comprehensive perspective on helios.TALK's positioning in relation to decentralized alternatives.

 Comment 3: *Third, the paper lacks a discussion and/or evaluation of the performance and scalability of the framework w.r.t. the user number increase. Specifically, how does the transaction throughput increase if the number of users grows N times? Are there any sensitive delays or storage limitations? These aspects are crucial for determining the practicality of the proposed solution.*  

 Response 3: In terms of scalability, all integrated modules within helios.TALK are designed to process in a manner directly proportional to the number of connections on each user's device, including the machine learning components. Additionally, the communication service simply relays messages without processing or storing them, resulting in processing costs aligned with the number of messages transmitted per unit of time. It should be stressed that the essence of helios.TALK lies in the versatility and flexibility it offers, where the choice of communication protocols can vary. The developed modules do not enforce a particular communication framework; instead, they allow for integration with various decentralized communication protocols. Hence, we regard evaluating the scalability of the proposed framework as beyond the scope of this paper.
